# Linking maize root size to phosphorus acquisition and productivity in diversified cropping systems

**DOI:** 10.3389/fpls.2026.1876721

**Published:** 2026-07-13

**Authors:** Ai Zhan, Di Huang, Ruirui Zhu, Xuhui Yang, Shuo Liu, Yinglong Chen

**Affiliations:** 1 State Key Laboratory of Soil and Water Conservation and Desertification Control, College of Soil and Water Conservation Science and Engineering, Northwest A&F University, Xian Yang, China; 2 College of Natural Resources and Environment, Northwest A&F University, Xian Yang, China; 3 Institute of Farmland Irrigation, Chinese Academy of Agriculture Sciences/Shangqiu, Agro-ecological System, National Observation and Research Station, Xinxiang, Henan, China; 4 The UWA Institute of Agriculture, School of Agriculture and Environment, The University of Western Australia, Perth, WA, Australia

**Keywords:** intercropping, land equivalent ratio, mixed-cropping, N and P accumulation, root architecture

## Abstract

**Introduction:**

Intercropping and mixed-cropping of plant varieties can increase yields; however, the impacts of root size and morphology on nutrient uptake and plant growth in these systems remain unclear.

**Methods:**

Two maize varieties with small root system (Shengrui 99, M1) and large root system (Zhongke 11, M2) were intercropped with soybean (D76–1609), and also grown under mixed-cropping conditions, with two phosphorus (P) levels (low P: 2.97 mg P kg^-1^ soil, LP, and high P: 175 mg P kg^-1^ soil, HP) under controlled environment.

**Results and discussion:**

Results showed that both intercropping and mixed-cropping conferred advantages over monoculture, with land equivalent ratios (LER) ranged from 1.87 to 2.53. The advantage of intercropping was greater under LP conditions, average 8.65% higher, while mixed-cropping performed better under HP conditions, increased by 26.20%. Nutrient absorption equivalent ratios were also greater than 1 in inter- and mixed-cropping systems. Intercropping with soybean enhanced maize root length under LP, with M2 showing a more pronounced response. Specific nutrient uptake per unit root length and nitrogen (N) and P competition ratios were significantly influenced by P supply and planting pattern (*P<0.05*). Notably, under LP conditions, M2 exhibited stronger competitive ability for P, while M1 showed greater N competitiveness.

**Conclusion:**

These results suggest that genetic improvement of maize root size could enhance plant growth and improve resource competitiveness in intercropping and mixed-cropping systems. Furthermore, depending on P availability, larger root systems in maize could increase overall productivity in intercropping and mixed-cropping systems.

## 1 Introduction

Phosphorus (P) is an essential macronutrient for plant growth and development, laying a pivotal role in increasing crop yield (Kochian, 2012; Cordell and White, 2014). Unfortunately, due to its high fixation and low mobility in soil, this essential macronutrient is often highly unavailable for uptake by plant roots (Pereira and Castro, 2014; Achat et al., 2016; Hufnagel et al., 2024). Approximately half of the world’s agricultural land lacks adequate available P to support optimal crop production (Lynch, 2011). Hence, improving plant ability to capture P from low-P soils—through strategies such as optimizing root architecture (Lynch, 2013) or improving cultivation patterns (Kroon, 2007)—is of utmost importance for ensuring global food security.

Intercropping is a traditional multiple cropping system in which two or more crop species are grown simultaneously in the same field for a significant portion of their growth cycle (Tang et al., 2021; Wang et al., 2024). Maize–soybean intercropping represents a classic and widely studied example of such a system (Kermah et al., 2017). In this system, above- and below-ground interactions between intercropped species resulted in the enhanced nutrient uptake and yield increment (Li et al., 2021; Zhang et al., 2022). However, below-ground interactions far less investigated than above-ground traits, even though many studies reported that belowground root interspecific interactions are more pronounced than aboveground interactions in the intercropping system (Duchene et al., 2017; Streit et al., 2019). Plant root is an important organ for anchorage, water and nutrient uptake. Its spatial distribution within the soil can influenced by various abiotic and biotic factors such as nutrient availability and intra- and interspecific competitions (Lin et al., 2024; Wang et al., 2026). For instance, maize roots extend toward the soybean side and proliferate beneath soybean plants in the maize-soybean intercropping systems, whereas soybean roots are largely confined to the topsoil and show limited growth toward the maize side (Zhang et al., 2022). Faster root growth has been reported in cereal-legume intercropping, associated with higher nutrient uptake and shoot biomass accumulation in maize (Schwerdtner and Spohn, 2021). These studies on the intercropping were only focused on single crop varieties, lacking research on intercropping effects associated with distinct genotype-specific root crops.

Recently, a novel planting pattern known as mixed-cropping—simultaneously cultivating multiple cultivars of the same species—has gained increasing attention among researchers (Reiss and Drinkwater, 2018). Compared to traditional intercropping systems, mixed-cropping retains ecological and agronomic benefits while offering greater practicality in sowing, field management, and harvesting, making it particularly suitable for mechanized agricultural systems (Barot et al., 2017). Consequently, this approach has been widely adopted by farmers in regions such as China, East Germany, Poland, Denmark, and Switzerland (Wolfe, 1985; Zhu et al., 2000; Malézieux et al., 2009). A meta-analysis of 94 studies has demonstrated that mixed-cropping systems consistently achieve higher yields and greater yield stability, especially under biotic and abiotic stress conditions (Reiss and Drinkwater, 2018). A study on eight maize varieties with varying growth periods and plant types revealed that mixed-cropping not only enhanced nutrient uptake and yield but also improved productivity stability compared to monoculture (Wu, 2020). Despite these advances, existing research has largely focused on the effects of factors such as plant type, growth stage, and species composition on disease control, nutrient acquisition, and yield (Reiss and Drinkwater, 2018; Zhu et al., 2000). Little attention has been paid to the role of root architectural variation among cultivars in shaping root distribution, nutrient uptake efficiency, and overall plant growth in mixed-cropping systems.

Therefore, in this study, we selected two maize varieties—Shengrui 99 (with a small root system) and Zhongke 11 (with a large root system)—based on our previous screening of root architectural traits across 174 maize genotypes. These two varieties exhibit distinct root architectures while maintaining comparable aboveground biomass. We examined how contrasting root systems influence root distribution, nitrogen (N) and phosphorus (P) uptake, and plant growth under three planting patterns: maize–soybean intercropping, mixed-cropping of the two maize varieties, and monocultures of maize and soybean. We hypothesised that (1) intercropping and mixed-cropping will alter plant growth and nutrient uptake compared with monoculture; and (2) root system size influences the advantages of intercropping and mixed-cropping.

## 2 Materials and methods

### 2.1 Plant and soil materials

Two maize (*Zea mays* L.) varieties (small-rooted Zhongke 11, M1, and large-rooted Shengrui 999, M2) were selected from our early phenotyping study for root traits involving 174 genotypes. The two varieties differed in root size and morphology but had similar shoot biomass (**Supplementary Table 1**; Qiao et al., 2019; Liu et al., 2021). Soybean (*Glycine max* (L.) Merr.) variety D76–1609 was selected from a root study involving 171 genotypes popularized in the Yangtze and Huaihe River regions, eastern China (Liu et al., 2021). Silt loam soil (Eumorthic Anthrosols) was collected from the top 10 cm layer of a farm in Yangling, Shaanxi province, China, and sieved through a 2 mm sieve after air dried. It was then mixed with coarse river sand at a ratio of 2:1 (soil: sand, w/w). The properties of mixed medium were 4.86 g kg^-1^ organic matter, 0.31 g kg^-1^ total N, 24.0 mg kg^-1^ available N, 2.97 mg kg^-1^ available P, 169 mg kg^-1^ available K, and pH 8.4. Rhizobox (15 cm length, 3 cm wide, and 60 cm height), with one side made by clear perplex glass and covered by a black plastic sheet during the experiment, were used in this study. Each rhizobox was filled with 3.3 kg of the mixed soil. Before filling, a 2 cm layer of gravel was placed at the bottom of each rhizobox to prevent soil from blocking the drainage hole. Basic fertilizers (200 mg N kg^–1^ soil as urea and 200 mg K kg^–1^ soil as KCl). Fertilizers were thoroughly mixed with the soil before filling to ensure homogeneous distribution.

### 2.2 Experimental design and growth conditions

The experiment was conducted with two P supply levels and six planting patterns. Each treatment was replicated four times. The two P supply levels were LP (2.97 mg P kg^-1^ soil), and HP (175 mg P kg^-1^ soil). Six planting patterns were soybean monoculture (S), maize monoculture (two maize varieties: M1, M2), maize–soybean intercropping (M1S, M2S), and mixed-cropping of the two maize varieties (M1M2).

Maize and soybean seeds were surface sterilised in 10% H_2_O_2_ for 10 min, rinsed thoroughly with distilled water, and soaked overnight in a saturated CaSO_4_ solution. Each rhizobox contained two plants spaced 10 cm apart. Three seeds of each species were sown in a hole near the perplex glass side at a depth of 2 cm. Seven days after sowing, seedlings were thinned to one plant per hole; therefore, each rhizobox contained two plants in total.

Soil water content in each rhizobox was measured using a portable TDR device (time domain reflectometry, Campbell Scientific Australia Pty. Ltd., 15 Townsville Artarmon, New South Wales, Australia). Soil water content was maintained at approximately 70 ± 5% of water-holding capacity. Rhizoboxes were placed in a glasshouse at Northwest A&F University, Yangling (34°16′ N, 108° 4′ E). The average daily temperature during the experimental period was approximately 25/15 °C (day/night).

### 2.3 Sampling and analysis

Plants were harvested 25 days after sowing. The number of visible roots via the glass wall was recorded every week after 10 days of sowing until harvest. For each measurement, transparent plastic film (A4 paper size) was covered in the perplex glass side after removed the black plastic sheet, and the visible new roots was manually traced by black marker. Root length was obtained by scanning the plastic film (Epson Perfection V800, Long Beach, CA, USA).

At harvest, each rhizobox was laid horizontally on a flat bench, shoots were severed at the soil surface, and shoot dry mass was measured after drying at 75°C for 72 h. Roots of the two plants in each rhizobox were then manually separated. Root subsamples were collected from five soil depth sections (0–10 cm, 10–20 cm, 20–30 cm, 30–40 cm, 40–60 cm), and thoroughly washed with water.

Root morphology was analysed using a scanner (EPSON Perfection V800, Long Beach, CA, USA). The scanned root images were processed to determine total root length using WinRHIZO software (v2009, Regent Instruments Inc. Quebec, QC, Canada). Root dry mass for each section was recorded after image analysis. Specific N and P uptake per unit root length (SNU, SPU) were calculated as shoot N or P accumulation divided by total root length. The root-to-shoot ratio (R/S) was calculated as total root dry mass divided by shoot dry mass.

Oven-dried shoots and roots were ground, and N and P concentrations were determined using H_2_SO_4_–H_2_O_2_ digestion method. Tissue N and P contents were calculated from the respective nutrient concentrations and the dry mass of each tissue.

Land equivalent ratio (LER) was calculated using the following equation (Rao and Willey, 1980) and Yuan et al. (2017):


$$\text{LER}=\frac{{\text{Y}}_{1}}{{\text{Y}}_{1\text{M}}}+\frac{{\text{Y}}_{2}}{{\text{Y}}_{2\text{M}}}$$

Where Y_1_ and Y_2_ were shoot dry mass of maize and/or soybean in intercropping or mixed-cropping system, Y_1M_ and Y_2M_ were shoot dry mass in monoculture. When LER>1, it means that the intercropping or mixed-cropping system has an advantage over monoculture; when LER<1, it means that the shoot dry mass of the intercropping or mixed-cropping system was disadvantage.

N and P absorption equivalent ratio (ER_P_ and ER_N_) was calculated as described by Facelli et al. (2010):


$$\text{ER}=\frac{{\text{P}}_{1}}{{\text{P}}_{1\text{M}}}+\frac{{\text{P}}_{2}}{{\text{P}}_{2\text{M}}}$$

Where P_1_ and P_2_ were N or P accumulation of maize and/or soybean in intercropping, or the two maize genotypes (M1 and M2) in the mixed-cropping system. P_1M_ and P_2M_ were N or P accumulation of maize and/or soybean in monoculture. When ER >1, it means that the intercropping or mixed-cropping system has a N or P absorption advantage than the monoculture; when ER <1, it means that N or P absorption of the intercropping or mixed-cropping system is at a disadvantage.

Nutrient competition ratio (CR) was calculated as described by Morris and Garrity (1993):


$$\text{CR}=(\frac{{\text{P}}_{1}}{{\text{P}}_{1\text{M}}})/(\frac{{\text{P}}_{2}}{{\text{P}}_{2\text{M}}})$$

Where P_1_ and P_2_ were N or P accumulation of maize and/or soybean in intercropping, or the two maize genotypes (M1 and M2) in mixed-cropping, P_1M_ and P_2M_ were N or P accumulation of maize and/or soybean in monoculture. When CR>1, it indicates that maize has stronger nutritional competitiveness than soybean, or M2 has stronger nutritional competitiveness than M1; when CR<1, it indicates that maize has weaker nutritional competitiveness than soybean, or M2 has weaker nutritional competitiveness than M1.

### 2.4 Statistical analysis

Analysis of variance (ANOVA) was conducted using SAS statistical software (SAS version 9.4, USA). Least significant differences (LSD) were used to separate treatment means at the 5% level. The plant growth and root length per rhizobox were subjected to two-way ANOVA to assess the effects of planting patterns, P supply, and their interaction.

## 3 Results

### 3.1 General overview of plant growth

Phosphorus (P) supply had significant effects on total nitrogen (N) accumulation (TNA) and total P accumulation (TPA) (**Table 1**). Planting pattern also showed significant, and in some cases highly significant, effects on total dry mass (TDM), total root length (TRL), TNA, TPA. The interaction between P supply and planting pattern had strong effects on TRL, TNA, and TPA.

**Table 1:** Total dry mass, total P accumulation, and total root length per rhizobox as influenced by P fertilizer application (F, low P: 2.97 mg P kg^-1^ soil, refers to LP, high P: 175 mg P kg^-1^ soil, refers to HP) and planting patterns (PP, M, maize monoculture; S, soybean monoculture; MS, maize-soybean intercropping; M1M2, mixed cropping of two maize genotypes; M1, Shengrui 999; M2, Zhong ke 11) for 25 days after sowing.

Fertilizer P rates (F)	Planting patterns (PP)	Total dry mass (g rhizobox^–1^)	Total N accumulation (mg rhizobox^-1^)	Total P accumulation (mg rhizobox^-1^)	Total root length (m rhizobox^–1^)
Low P	S	0.40 f	20.93 cde	1.83 de	4.45 e
	M1	0.73 bc	20.33 cde	1.58 ef	12.24 ab
	M2	0.70 bcd	19.46 def	1.20 g	14.21 a
	M1S	0.57 e	17.03 f	1.22 g	8.38 cd
	M2S	0.70 bcd	23.88 ab	2.08 d	10.67 b
	M1M2	0.70 bcd	20.91 cde	1.31 fg	10.42 bc
High P	S	0.44 f	20.50 cde	2.08 d	4.50 e
	M1	0.73 bc	22.79 bc	2.81 ab	11.15 ab
	M2	0.76 ab	18.95 ef	2.81 ab	13.22 a
	M1S	0.60 de	21.89 bcd	2.62 bc	8.15 d
	M2S	0.63 cde	20.90 cde	2.46 c	8.38 cd
	M1M2	0.86 a	25.72 a	3.11 a	13.04 a
ANOVA					
F		3.91NS	7.23*	363.34**	0.11NS
PP		25.82***	6.82**	4.44**	44.24***
F × PP		2.20NS	6.57**	20.34***	2.49*

For each column, mean data (*n* = 4) followed by different lowercase letters are significantly different at the 5% level by LSD. *P* ≤ 0.05, *, *P* ≤ 0.01 **, *P* ≤ 0.0001***, NS, non-significant.

P supply increased TNA in small rooted maize-soybean intercropping system (M1S) and mixed-cropping of the two maize varieties (M1M2) by 28.54% and 23.00%, respectively, but decreased it in big rooted maize-soybean intercropping system (M2S) by 12.48%. P supply also markedly enhanced TPA in all treatments except soybean monoculture (S). Under low P conditions (LP), compared with maize monoculture (M1, M2), M1S significantly decreased TDM by 21.92%, but showed no significant difference compared with M2S and M1M2. TNA and TPA in M1S were lower than in S and M1, by 18.63% and 33.33%, and 16.23% and 22.78%, respectively. TPA in M2S showed no significant difference from S but was substantially higher than in M2, by 73.33%.

Under high P supply (HP) conditions, compared with maize monoculture, TDM in both M1S and M2S was significantly decreased by 17.81% and 17.11%, respectively, whereas TDM in M1M2 increased by17.81% and 13.16%, respectively. TNA in M2S was higher than in both S and M2 by 14.09% and 17.46%, respectively. TPA in M1S showed no significant difference from M1 but was markedly higher than S by 25.96%. TPA in M2S was significantly higher than S but significantly lower than M2.

Under both LP and HP conditions, TRL in M1S was significantly higher than in S but significantly lower than in M1. TRL in M2S was significantly higher than in both S and M2 under both LP and HP. Under LP, TRL in M1M2 showed no significant difference from M1 but was lower than M2.

**Figure 1:**
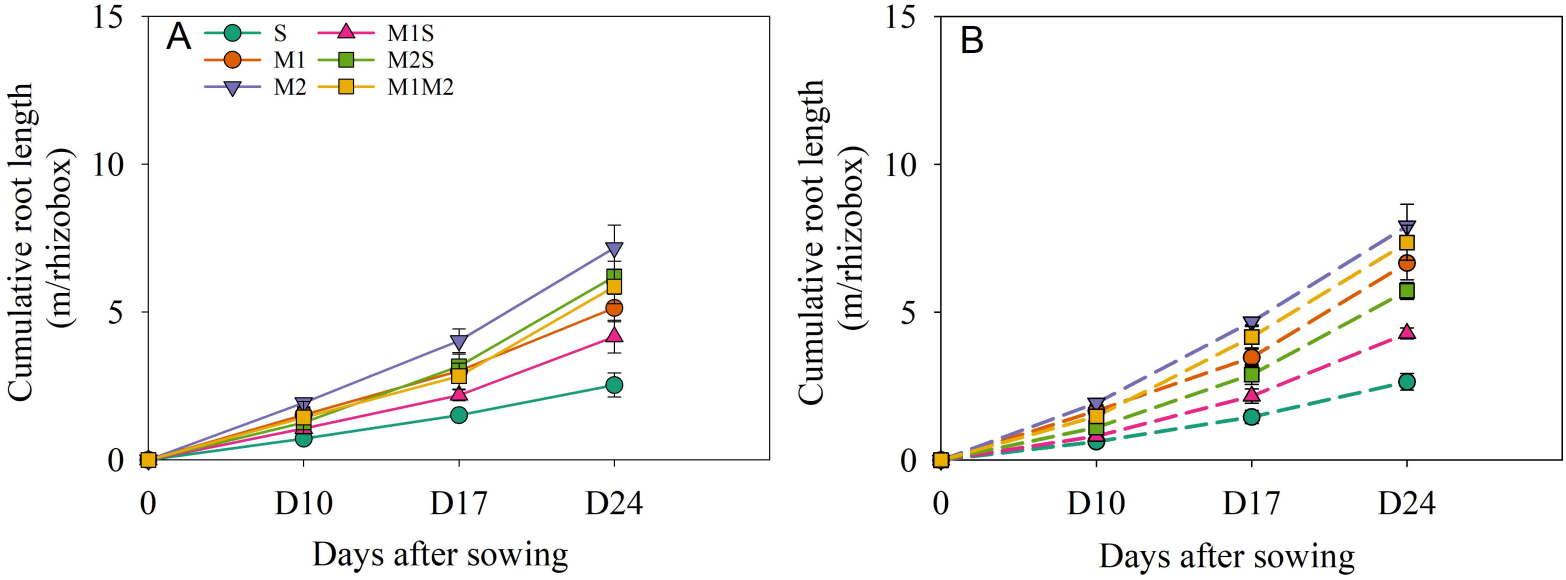
Cumulative root length per rhizobox of six planting patterns in low P **(A)** and high P **(B)** conditions. M1, maize genotype Shengrui 999 monoculture; M2, maize genotype Zhong ke 11 monoculture; S, soybean monoculture; M1S, maize gentype M1-soybean intercropping; M2S, maize gentype M2-soybean inter cropping; M1M2, mixed cropping of two maize genotypes. Values are presented as means (*n* = 4) and bars indicate standard errors.

Accumulated root length per rhizobox was influenced by planting patterns (**Figure 1**). Under LP conditions, among six planting patterns, M2 indicated the highest accumulated root length, while S showed the lowest accumulated root length across all three sampling times. Before 17 DAS, accumulated root length showed no difference among the treatments of M1, M2S and M1M2. After that, accumulated root length in M2S started increase and finally higher than M1 and M1M2. Under HP conditions, accumulated root length was indicated the highest in M2, while S showed the lowest through the growth period.

### 3.2 Shoot and root dry mass of maize and soybean

**Figure 2:**
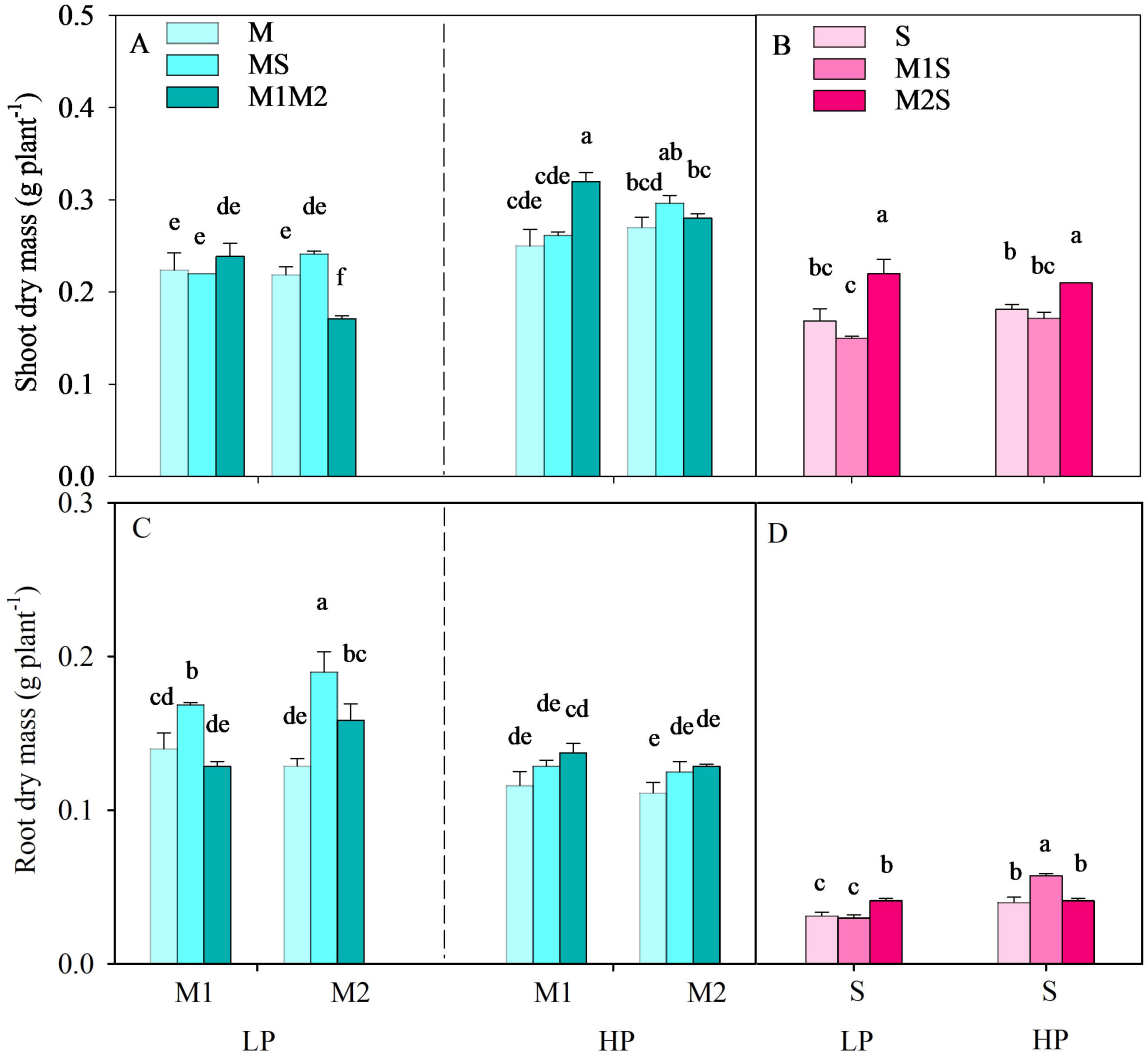
Shoot dry mass and root dry mass of maize **(A, C)** and soybean **(B, D)** in response to P fertilizer application rates (low P: 2.97 mg P kg^-1^ soil, refers to LP, high P: 175 mg P kg^-1^ soil, refers to HP) and planting patterns (M, maize monoculture; S, soybean monoculture; MS, maize-soybean intercropping; M1M2, mixed cropping of two maize genotypes; M1, Shengrui 999; M2, Zhong ke 11). Values are presented as means (*n* = 4) and bars indicate standard errors. Within each plot, means accompanied by different letters are significantly different at *P* ≤ 0.05. Asterisks indicate significant differences between HP and LP in the same planting pattern.

**Figure 3:**
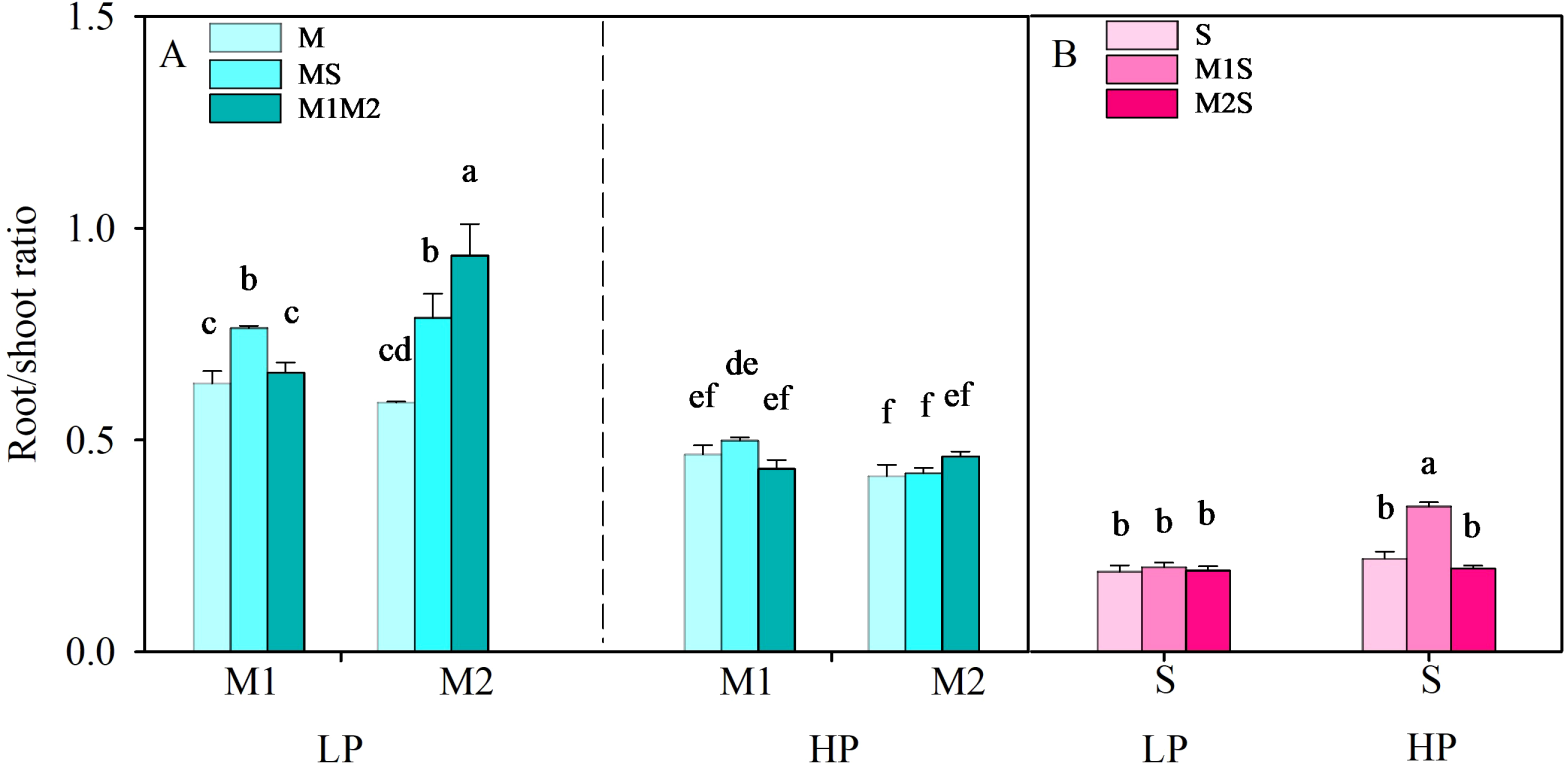
Root shoot ratio of maize **(A)** and soybean **(B)** in response to P fertilizer application rates (low P: 2.97 mg P kg^-1^ soil, refers to LP, high P: 175 mg P kg^-1^ soil, refers to HP) and planting patterns (M, maize monoculture; S, soybean monoculture; MS, maize-soybean intercropping; M1M2, mixed cropping of two maize genotypes; M1, Shengrui 999; M2, Zhong ke 11). Values are presented as means (*n* = 4) and bars indicate standard errors. Within each plot, means accompanied by different letters are significantly different at *P* ≤ 0.05. Asterisks indicate significant differences between HP and LP in the same planting pattern.

P supply significantly increased maize shoot dry mass (SDM), with an average increase of 27.69%, but significantly decreased root dry mass (RDM) and the root-to-shoot ratio (R/S), by an average of 18.31% and 38.36%, respectively (**Figures 2A, C**, **3A**; **Table 2**). Under both LP and HP conditions, planting pattern significantly affected SDM, RDM, and R/S (**Figures 2**, **3**; **Table 2**).

**Table 2:** The F value of three-way (maize) or two-way (soybean) analysis of variance of shoot dry mass (SDM), root dry mass (RDM), root/shoot ratio (R/S ratio), shoot N accumulation (SNA), shoot P accumulation (SPA), root N accumulation (RNA), root P accumulation (RPA), specific N uptake (SNU), and specific P uptake (SPU) in fertilizer P application (F), genotypes (G), and the planting patterns (PP).

Crop	Source of Variation	SDM	RDM	R/S Ratio	SNA	SPA	RNA	RPA	SNU	SPU
Maize	F	99.27***	48.36***	227.48***	106.85***	793.00***	109.88***	7.71**	94.69***	1016.80***
	G	6.75**	9.90***	14.61***	17.51***	0.16NS	39.50***	13.50***	6.27**	3.21*
	PP	19.75***	0.04NS	7.30**	62.84***	5.78*	1.33NS	14.49**	10.58**	0.28NS
	F×G	6.99**	10.52***	16.89***	1.80NS	1.75NS	7.61**	10.84***	18.11***	10.23***
	F×PP	0.87NS	0.00NS	1.03NS	0.10NS	17.32***	1.59NS	4.24*	12.67**	7.56**
	G×PP	4.33*	7.16**	6.43**	23.10***	0.81NS	5.86**	1.00NS	50.67***	14.37***
	P×G×PP	4.95**	6.38**	1.57NS	16.14***	6.67**	3.71*	9.39**	35.57***	8.08**
Soybean	F	0.40NS	58.33***	55.71***	1.54NS	75.28***	2.61NS	16.51**	0.09NS	22.44***
	PP	23.46***	9.79**	38.90***	20.45***	37.55***	8.18**	5.23**	3.78*	0.92NS
	F×PP	1.02NS	27.03***	31.45***	11.34***	19.36***	4.83*	2.59*	0.66NS	0.78NS

*P* ≤ 0.05, *, *P* ≤ 0.01 **, *P* ≤ 0.0001***, NS, non-significant.

Under LP conditions, compared with monoculture, maize–soybean intercropping significantly increased RDM and R/S of M1 and M2 in MS, with average increases of 20.54% and 47.57%, and 20.71% and 24.00%, respectively. Mixed-cropping of the two maize varieties (M1M2) had no significant effects on SDM, RDM, or R/S of the small-rooted maize variety M1, but significantly increased RDM and R/S of the large-rooted maize variety M2 by 23.30% and 58.92%, respectively.

Under HP conditions, maize–soybean intercropping showed no significant differences compared with maize monoculture in SDM, RDM, and R/S. Compared with M1 monoculture, mixed-cropping of the two maize varieties significantly promoted SDM of the small-rooted maize variety M1 by 28.00%.

For soybean, P supply significantly increased SDM, RDM, and R/S, with average increases of 4.41%, 35.37%, and 30.98%, respectively (**Figures 2B, D**, **3B**; **Table 2**). Soybean growth was significantly affected by the intercropped maize variety (**Table 2**). Under LP conditions, intercropping with the large-rooted maize variety M2 significantly promoted soybean SDM and RDM by 30.37% and 32.00%, respectively (**Figures 2**, **3**; **Table 2**). Under HP conditions, intercropping with M2 significantly promoted soybean SDM by 15.86%.

### 3.3 Nutrient accumulation of maize and soybean

**Figure 4:**
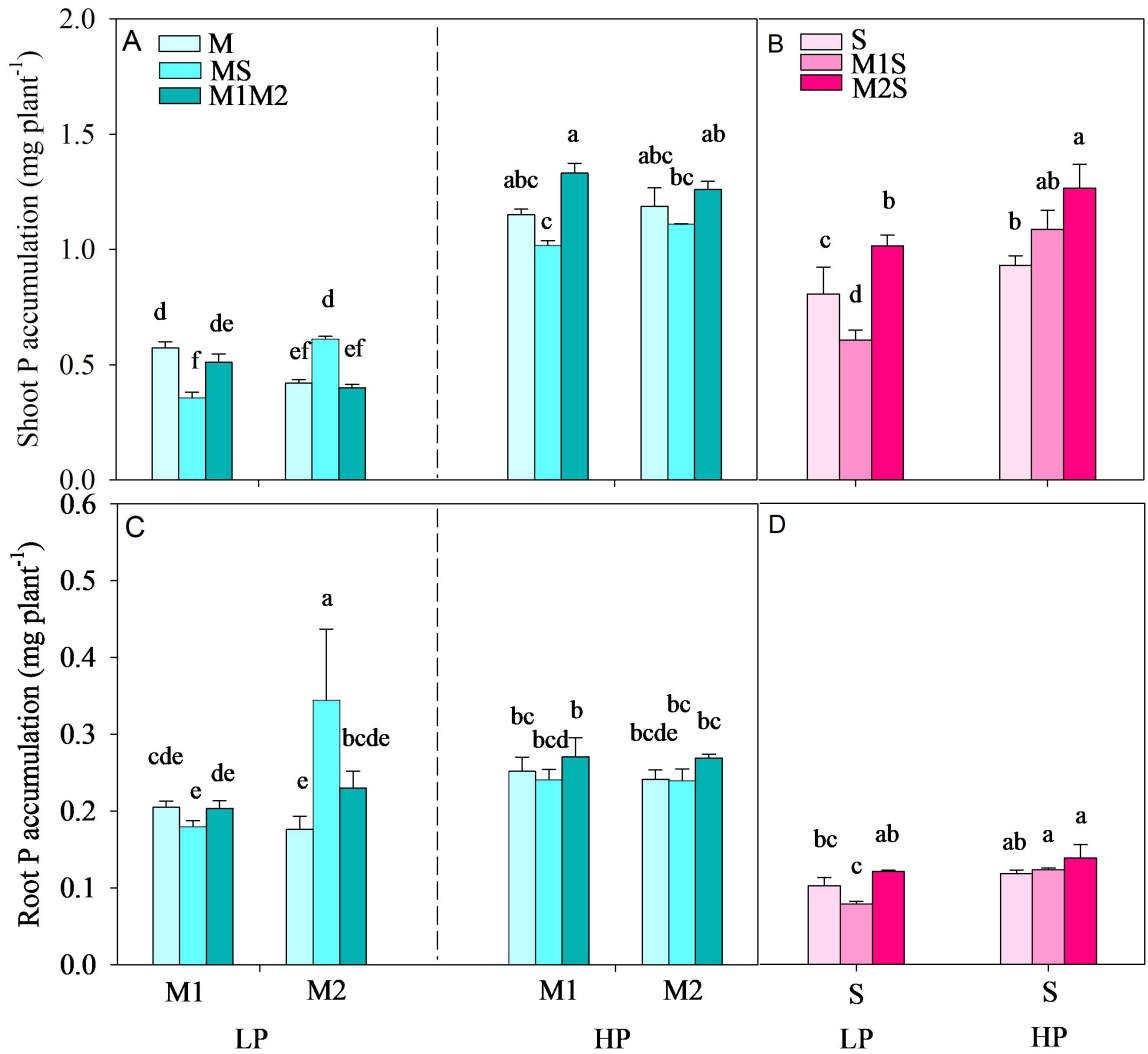
Shoot and root N accumulation of maize **(A, C)** and soybean **(B, D)** in response to P fertilizer application rates (low P: 2.97 mg P kg^-1^ soil, refers to LP, high P: 175 mg P kg^-1^ soil, refers to HP) and planting patterns (M, maize monoculture; S, soybean monoculture; MS, maize-soybean intercropping; M1M2, mixed cropping of two maize genotypes; M1, Shengrui 999; M2, Zhong ke 11). Values are presented as means (*n* = 4) and bars indicate standard errors. Within each plot, means accompanied by different letters are significantly different at *P* ≤ 0.05. Asterisks indicate significant differences between HP and LP in the same planting pattern.

Planting pattern and P supply significantly influenced shoot N accumulation (SNA) in both maize and soybean (**Figure 4**, **Table 2**). P supply notably increased maize SNA and soybean root N accumulation (RNA) by 27.62% and 8.00%, respectively, but resulted in a decrease in maize RNA (**Figure 4**, **Table 2**).

Under LP conditions, there were no significant differences in SNA and RNA between monocultures of the two maize varieties. Intercropping with soybean significantly reduced the SNA of the small-rooted maize variety M1 by 14.30%, while significantly increasing the RNA of the large-rooted maize variety M2 by 36.18%. M1M2 considerably enhanced the SNA of M1 and the RNA of M2, but significantly suppressed the SNA of M2.

In contrast to maize, P supply had no significant effect on soybean SNA and RNA (**Figures 4B, D**; **Table 2**). Soybean SNA was influenced by the intercropped maize variety, particularly under LP conditions. When intercropped with the small-rooted maize variety M1, soybean SNA and RNA significantly decreased by 23.84% and 25.80%, respectively. However, soybean SNA increased by 24.38% when intercropped with the large-rooted maize variety M2.

**Figure 5:**
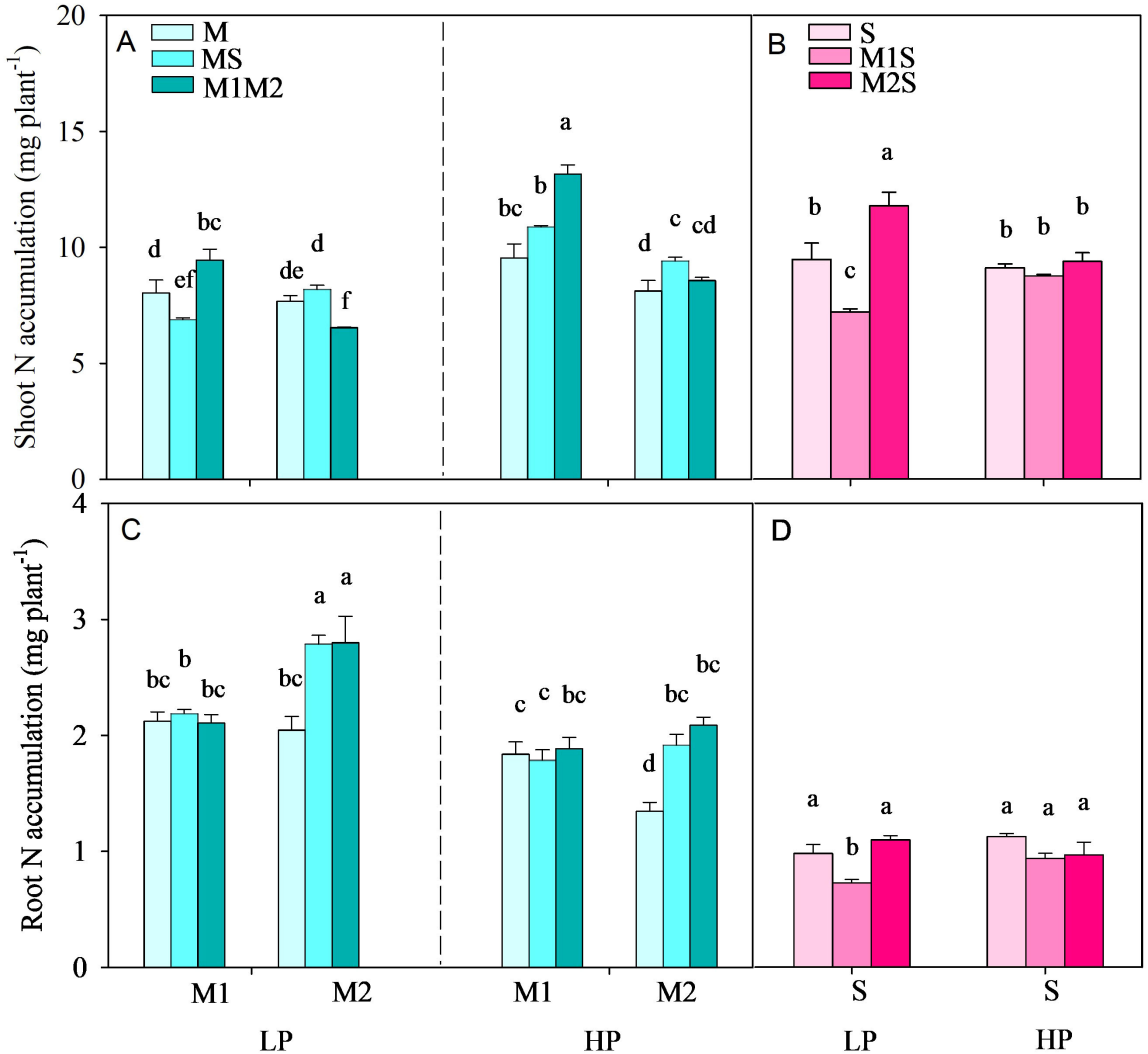
Shoot and root P accumulation of maize **(A, C)** and soybean **(B, D)** in response to P fertilizer application rates (low P: 2.97 mg P kg^-1^ soil, refers to LP, high P: 175 mg P kg^-1^ soil, refers to HP) and planting patterns (M, maize monoculture; S, soybean monoculture; MS, maize-soybean intercropping; M1M2, mixed cropping of two maize genotypes; M1, Shengrui 999; M2, Zhong ke 11). Values are presented as means (*n* = 4) and bars indicate standard errors. Within each plot, means accompanied by different letters are significantly different at *P* ≤ 0.05. Asterisks indicate significant differences between HP and LP in the same planting pattern.

P supply significantly enhanced maize P accumulation (SPA) and root P accumulation (RPA), by approximately 145.79% and 13.03%, respectively (**Figures 5A, C**; **Table 2**). Under LP conditions, planting patterns significantly influenced maize SPA and RPA (**Figures 5A, C**; **Table 2**). SPA in M1 monoculture was significantly higher than in M2, while RPA did not differ significantly between them. Intercropping with soybean significantly decreased SPA of M1 by 37.82%, but markedly increased SPA and RPA of M2 by 45.54% and 94.77%, respectively. There were no significant effects of mixed-cropping on either SPA or RPA. Under HP conditions, maize SPA and RPA were not significantly affected by planting patterns.

Soybean SPA and RPA were significantly influenced by P supply, planting patterns, and their interaction (**Figures 5B, D**; **Table 2**). P supply increased soybean SPA and RPA by 35.16% and 26.03%, respectively. Soybean SPA and RPA were also affected by the intercropped maize variety. Under LP conditions, intercropping with the small-rooted maize variety M1 reduced soybean SPA by 24.81%, whereas intercropping with the large-rooted maize variety M2 increased soybean SPA by 25.88%. Under HP conditions, intercropping with M1 had no significant effect on soybean SPA, while intercropping with M2 significantly increased soybean SPA by 35.96%.

### 3.4 Root distribution of maize and soybean

P supply and planting patterns affect maize and soybean total root length (TRL), root surface area (RSA), root volume (RV), and root diameter (RD) (**Table 3**). P supply decreased maize TRL, RSA, RV, and RD, except TRL, RSA and RV in M1M2, and RD in M2S. Under LP conditions, intercropping with soybean enhanced maize TRL, with increases of 6.27% for M1 and 19.10% for M2. Conversely, mixed-cropping of the two maize varieties reduced TRL, with decreases of 8.69% for M1 and 32.03% for M2. Under HP conditions, intercropping and mixed-cropping both promoted TRL in M1 by 10.70% and 16.13%, respectively. Soybean TRL was inhibited when intercropped with the small-rooted maize variety M1, with reductions of 15.87% and 11.88% under LP and HP conditions, respectively. Compare with monoculture, maize RSA was increased in the maize-soybean intercropping systems (M1S, M2S), but greatly decreased in the mixed cropping of the two maize varieties. Maize RSA, RV were slightly decreased in maize-soybean intercropping systems (M1S, M2S) under HP conditions, except TRL in M1S. RSA, and RV were greatly increased in the mixed cropping of the two maize varieties. Contrary to maize, P supply increased soybean RSA, except in M2S (**Table 3**). And compared with soybean monoculture, intercropping with small rooted maize genotype M1 greatly decreased soybean RSA, RV, and RD, but soybean RSA was greatly increased when intercropped with big rooted maize genotype M2, especially under LP conditions, increased by 11.20%.

**Table 3:** Total root length (TRL), root surface area (RSA), root volume (RV), and root diameter (RD) as influenced by P fertilizer application (low P: 2.97 mg P kg^-1^ soil, refers to LP, high P: 175 mg P kg^-1^ soil, refers to HP) and planting patterns (M, maize monoculture; S, soybean monoculture; MS, maize-soybean intercropping; M1M2, mixed cropping of two maize genotypes; M1, Shengrui 999; M2, Zhong ke 11) for 25 days after sowing.

Fertilizer P rates	Root growth	Maize						Soybean		
		M1	M1S	M1M2	M2	M2S	M1M2	S	M1S	M2S
LP	TRL (cm)	612.19 ± 30.87	650.58 ± 31.33	559.01 ± 59.23	710.56 ± 85.11	846.25 ± 47.18	482.99 ± 40.52	223.16 ± 20.59	187.74 ± 5.19	221.36 ± 19.97
	RSA (cm^-2^)	96.65 ± 8.52	97.07 ± 20.20	93.98 ± 12.00	102.47 ± 18.56	126.52 ± 21.34	77.35 ± 10.50	34.84 ± 5.60	23.94 ± 2.69	38.74 ± 2.90
	RV (cm^-3^)	1.26 ± 0.12	1.29 ± 0.25	1.28 ± 0.17	1.32 ± 0.23	1.74 ± 0.27	1.01 ± 0.16	0.45 ± 0.07	0.28 ± 0.03	0.49 ± 0.04
	RD (mm)	2.59 ± 0.09	2.74 ± 0.11	2.70 ± 0.21	2.08 ± 0.09	2.32 ± 0.24	1.81 ± 0.18	1.53 ± 0.26	0.98 ± 0.07	1.32 ± 0.15
HP	TRL (cm)	557.70 ± 48.07	617.43 ± 41.42	647.65 ± 46.03	660.77 ± 53.84	601.23 ± 89.74	656.45 ± 33.96	224.28 ± 5.47	197.64 ± 5.88	236.83 ± 4.81
	RSA (cm^-2^)	86.96 ± 12.14	83.38 ± 9.75	102.72 ± 10.45	94.98 ± 14.14	80.39 ± 14.19	104.61 ± 7.22	35.41 ± 5.38	27.71 ± 9.12	35.74 ± 4.57
	RV (cm^-3^)	1.14 ± 0.17	1.08 ± 0.13	1.34 ± 0.15	1.11 ± 0.16	0.98 ± 0.16	1.33 ± 0.09	0.45 ± 0.08	0.37 ± 0.06	0.49 ± 0.03
	RD (mm)	2.55 ± 0.10	2.48 ± 0.10	2.61 ± 0.10	2.30 ± 0.10	2.38 ± 0.15	2.26 ± 0.14	1.46 ± 0.02	1.13 ± 0.14	1.24 ± 0.15

Data are means (*n* = 4) ± SD.

**Figure 6:**
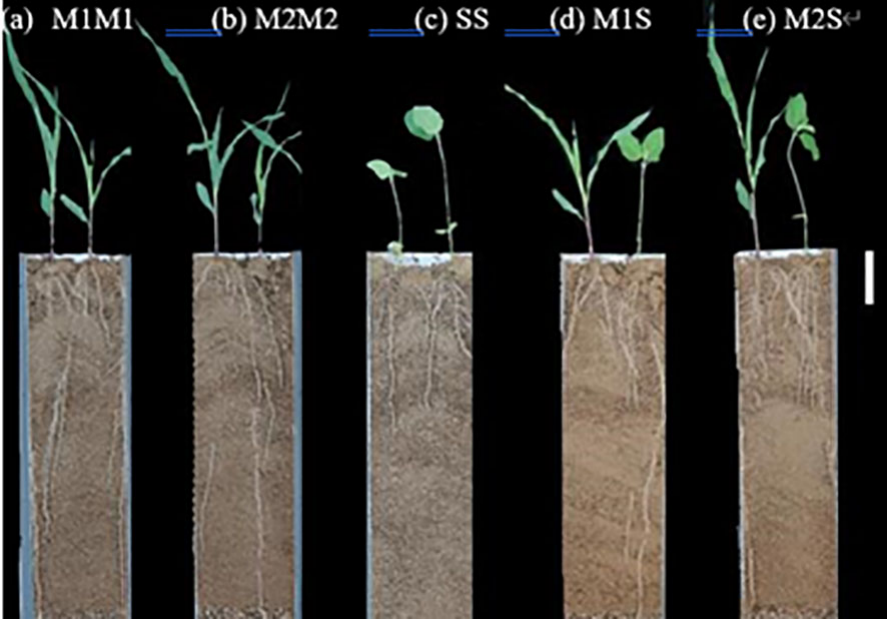
Maize and soybean grown in rhizobox at harvest under low P conditions.

Root growth of maize and soybean in the 0–60 cm soil profile was influenced by P supply and planting patterns (**Table 4**). Across 0–60 cm soil layers, maize and soybean RL generally decreased with increasing soil depth, with approximately 55.06% to 100% of roots distributed in the 0–20 cm layer (**Table 3**, **Figure 6**). Under LP conditions, intercropping with soybean suppressed M1 RL in the 0–20 cm soil layer, while enhancing RL of M2 across the 0–60 cm soil profile. Mixed-cropping inhibited RL of both M1 and M2 in the 0–60 cm soil profile. Conversely, under HP conditions, both intercropping and mixed-cropping enhanced RL of M1 across the 0–60 cm soil profile.

**Table 4:** Root distribution in 0–60 cm soil profile as influenced by P fertilizer application (low P: 2.97 mg P kg^-1^ soil, refers to LP, high P: 175 mg P kg^-1^ soil, refers to HP) and mixed cropping (M: maize monoculture; S: soybean monoculture; MS: Maize-soybean intercropping; M1M2: mixed cropping of two maize genotypes, M1, Shengrui 999, M2, Zhong ke 11) for 25 days after sowing.

Fertilizer P rates	Depth (cm)	Maize root length (cm)						Soybean root length (cm)			
		M1			M2			S			
		M1	M1S	M1M2	M2	M2S	M1M2		S	M1S	M2S
Low P	0-10	211.22 ± 15.82	203.73 ± 9.78	233.83 ± 26.44	240.42 ± 36.26	277.75 ± 46.33	199.12 ± 34.76		108.05 ± 21.84	116.90 ± 3.38	156.04 ± 2.18
	10-20	204.80 ± 18.26	186.30 ± 11.97	168.64 ± 27.58	223.20 ± 40.27	255.69 ± 41.39	149.87 ± 17.88		54.58 ± 14.39	70.85 ± 21.64	79.22 ± 6.83
	20-30	119.21 ± 14.56	142.64 ± 12.88	98.72 ± 17.26	153.16 ± 30.15	196.70 ± 59.33	75.00 ± 9.41		35.23 ± 1.26	–	23.63 ± 0.51
	30-40	49.79 ± 12.02	76.46 ± 4.28	43.14 ± 9.36	56.62 ± 13.24	100.45 ± 27.75	41.61 ± 5.55		25.29 ± 2.51	–	–
	40-60	31.49 ± 7.28	41.46 ± 5.41	19.58 ± 3.90	64.68 ± 4.30	48.51 ± 27.28	23.20 ± 1.30		–	–	–
High P	0-10	183.74 ± 20.59	212.19 ± 16.21	236.42 ± 16.37	213.06 ± 22.46	220.02 ± 44.48	232.67 ± 22.22		122.48 ± 22.01	117.83 ± 17.25	133.33 ± 31.96
	10-20	160.06 ± 23.38	146.96 ± 21.28	195.40 ± 18.81	155.11 ± 29.97	190.00 ± 62.59	176.30 ± 10.83		71.91 ± 5.41	49.92 ± 13.72	49.16 ± 15.49
	20-30	112.08 ± 27.80	143.20 ± 11.65	127.54 ± 14.63	150.79 ± 31.32	98.22 ± 7.64	141.81 ± 7.55		29.89 ± 1.66	–	21.70 ± 0.32
	30-40	71.21 ± 16.12	78.96 ± 10.29	60.45 ± 6.41	96.61 ± 22.03	53.22 ± 5.34	88.42 ± 17.04		–	–	–
	40-60	35.23 ± 6.78	36.12 ± 4.33	27.83 ± 4.73	53.07 ± 15.44	39.77 ± 3.15	34.50 ± 5.91		–	–	–

Compared with S monoculture, intercropping with M1 slightly increased soybean RL in the 0–20 cm soil layer under LP conditions and enhanced RL in the 0–30 cm soil layer under HP conditions. Intercropping with M2 increased soybean RL in the 0–20 cm soil layer under both LP and HP conditions.

### 3.5 Specific N uptake and specific P uptake

P supply significantly increased specific N uptake per unit root length (SNU) and specific P uptake per unit root length (SPU), by approximately 16.51% and 160.47%, respectively (**Tables 2**, **5**). Planting pattern had variable effects on SNU and SPU. Intercropping with soybean reduced SNU in both M1 and M2, except for M2 under HP conditions. Mixed-cropping improved SNU in both M1 and M2. Both intercropping with soybean and mixed-cropping decreased SPU in M1, while increasing it in M2.

**Table 5:** Specific P and N uptake of maize and soybean as influenced by P fertilizer application (low P: 2.97 mg P kg^-1^ soil, refers to LP, high P: 175 mg P kg^-1^ soil, refers to HP) and planting patterns (M, maize monoculture; S, soybean monoculture; MS, maize-soybean intercropping; M1M2, mixed cropping of two maize genotypes; M1, Shengrui 999; M2, Zhong ke 11) for 25 days after sowing. Data are means (*n* = 4) ± SD.

Maize	Fertilizer P rates	Genotype	M	MS	M1M2
Specific P uptake (mg m^-1^ root length)	LP	M1	0.10 ± 0.00	0.06 ± 0.00	0.09 ± 0.01
		M2	0.06 ± 0.01	0.07 ± 0.01	0.09 ± 0.01
	HP	M1	0.22 ± 0.03	0.21 ± 0.02	0.21 ± 0.02
		M2	0.18 ± 0.02	0.20 ± 0.03	0.19 ± 0.02
Specific N uptake (mg m^-1^ root length)	LP	M1	1.32 ± 0.08	1.07 ± 0.07	1.77 ± 0.22
		M2	1.11 ± 0.10	0.98 ± 0.06	1.40 ± 0.15
	HP	M1	1.77 ± 0.19	1.72 ± 0.13	2.08 ± 0.21
		M2	1.26 ± 0.12	1.70 ± 0.29	1.32 ± 0.07
Soybean			S	M1S	M2S
Specific P uptake (mg m^-1^ root length)	LP		0.40 ± 0.02	0.32 ± 0.01	0.47 ± 0.06
	HP		0.43 ± 0.01	0.48 ± 0.02	0.46 ± 0.02
Specific N uptake (mg m^-1^ root length)	LP		4.66 ± 0.23	3.86 ± 0.15	5.57 ± 0.17
	HP		4.24 ± 0.17	4.43 ± 0.15	3.99 ± 0.30

For soybean, under LP conditions, intercropping with M1 decreased both SNU and SPU, whereas intercropping with M2 enhanced both SNU and SPU (**Tables 2**, **5**). Under HP conditions, intercropping with M2 increased soybean SPU but decreased SNU. In contrast, intercropping with M1 simultaneously increased both soybean SNU and SPU.

### 3.6 Land equivalent ratio (LER), nutrient absorption equivalent ratio and nutrient competition ratio

LER under both LP and HP conditions across the three planting patterns were greater than 1, indicating the advantage of intercropping and mixed-cropping systems (**Table 6**). Specifically, LER values in M1S and M2S were higher under LP than under HP conditions, whereas M1M2 showed the opposite trend. This suggests that the advantage of intercropping was greater under P deficient conditions, while the advantage of mixed-cropping was greater under P-sufficient conditions. M2S consistently exhibited higher LER values compared with M1S under both LP and HP conditions. Under LP, LER in M1M2 was lower than in intercropping systems, whereas under HP, LER in M1M2 was higher than in intercropping systems.

**Table 6:** Land equivalent ratio (LER), N and P absorption equivalent ratio (ER_N_, ER_P_), and N and P competition ratio (CR_N_, CR_P_) as influenced by P fertilizer application (low P: 2.97 mg P kg^-1^ soil, refers to LP, high P: 175 mg P kg^-1^ soil, refers to HP) and planting patterns (M, maize monoculture; S, soybean monoculture; MS, maize-soybean intercropping; M1M2, mixed cropping of two maize genotypes; M1, Shengrui 999; M2, Zhong ke 11) for 25 days after sowing. Data are means (*n* = 4) ± SD.

Parameters	Fertilizer P rates	M1S	M2S	M1M2
LER	LP	1.99 ± 0.22	2.53 ± 0.25	1.87 ± 0.06
	HP	1.90 ± 0.07	2.26 ± 0.09	2.36 ± 0.13
ER_N_	LP	1.63 ± 0.05	2.32 ± 0.04	2.29 ± 0.06
	HP	2.05 ± 0.02	2.19 ± 0.12	2.44 ± 0.04
ER_P_	LP	1.36 ± 0.03	2.69 ± 0.04	1.82 ± 0.08
	HP	2.11 ± 0.02	2.13 ± 0.13	2.25 ± 0.14
CR_N_	LP	1.14 ± 0.08	0.88 ± 0.10	0.72 ± 0.03
	HP	1.14 ± 0.01	1.13 ± 0.05	0.78 ± 0.05
CR_P_	LP	0.86 ± 0.07	1.20 ± 0.09	1.11 ± 0.05
	HP	1.08 ± 0.04	0.81 ± 0.08	0.98 ± 0.17

The nitrogen absorption equivalent ratio (ER_N_) and phosphorus absorption equivalent ratio (ER_P_) of all three planting patterns were greater than 1 under both LP and HP conditions. M1S and M1M2 showed higher ER_N_ and ER_P_ under HP conditions than under LP conditions, whereas M2S showed the opposite trend. Under both LP and HP conditions, ER_N_ and ER_P_ in M2S and M1M2 were higher than those in M1S.

The nitrogen competition ratio (CR_N_) and phosphorus competition ratio (CR_P_) were significantly influenced by both P supply and planting patterns. Under LP conditions, CR_N_ was greater than 1 in M1S, but less than 1 in M2S and M1M2. Under HP conditions, CR_N_ exceeded 1 in both M1S and M2S. Under LP conditions, CR_P_ was greater than 1 in M2S and M1M2, but less than 1 in M1S. However, under HP conditions, CR_P_ showed the opposite trend: CR_P_ in M2S and M1M2 was less than 1, while it was greater than 1 in M1S.

## 4 Discussion

### 4.1 Intercropping and mixed-cropping improved maize growth and nutrient acquisition

**Figure 7:**
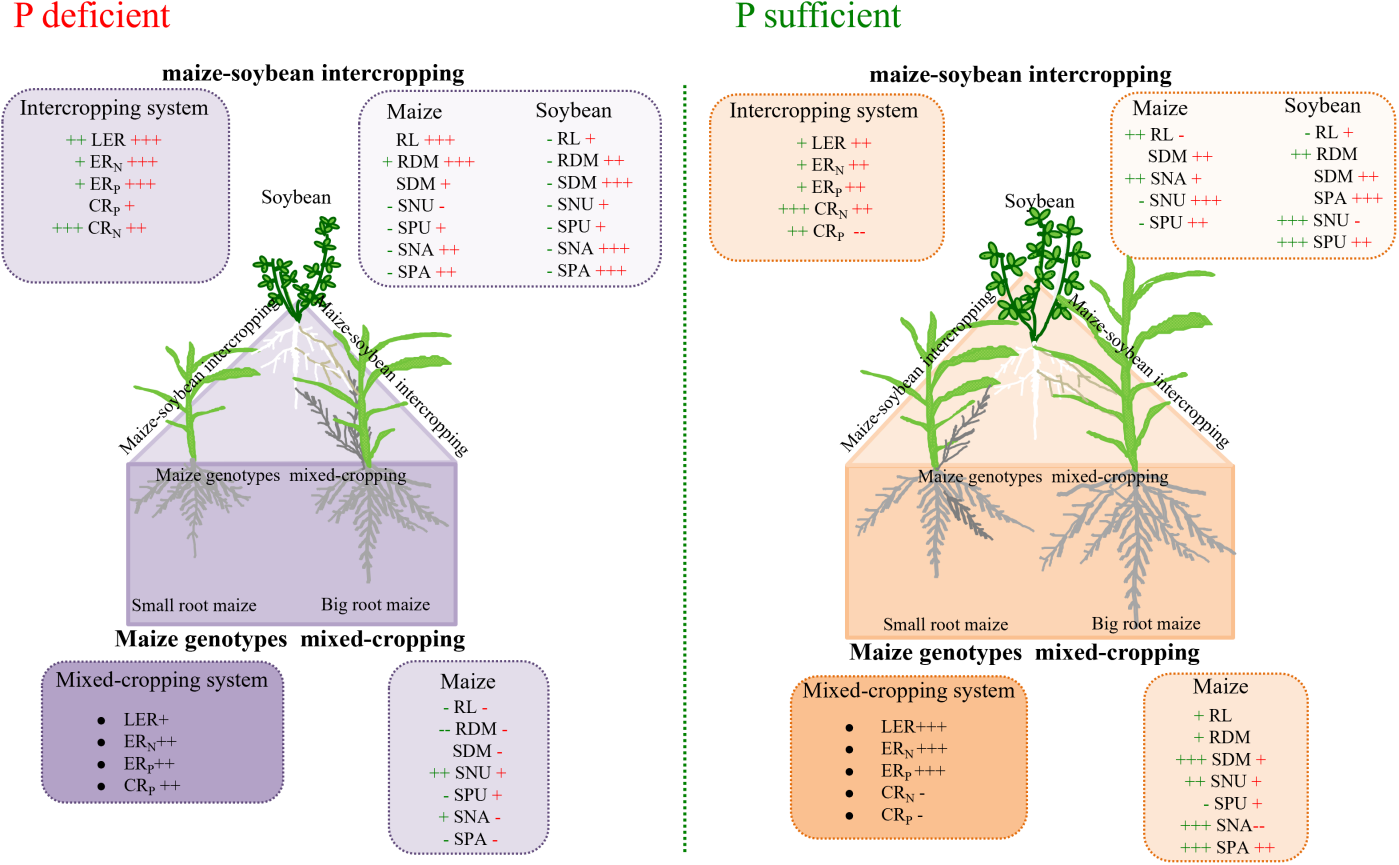
Schematic representation of different growth patterns under P deficient and P sufficient conditions in present study. Where LER, land equivalent ratio; ER_N_, N absorption equivalent ratio; ER_P_, P absorption equivalent ratio; CR_N_, N competition ratio; CR_P_, P competition ratio; RL, root length; SDM, shoot dry mass; RDM, root dry mass; SNA, shoot N accumulation; SPA, shoot P accumulation; SNU, specific N uptake; SPU, specific P uptake. +, positive effects; -, negative effects. The number of + or - represents the degree of effect; and for maize, the green color of – or + represents small rooted maize genotype Shengrui 999, the red color of – or + represents big rooted maize genotype Zhong ke 11; for soybean, the green color means the effects when intercropped with Shengrui 999, and red color means the effects when intercropped with Zhong ke 11.

Maize–soybean intercropping is a classical practice in traditional agricultural production systems (Kermah et al., 2017). This system enhances resource-use efficiency through complementary resource capture in both time and space, often leading to greater yields and economic returns compared with monocultures (Weerarathne et al., 2017; Li et al., 2021; Zhao et al., 2024). The land equivalent ratio (LER), a measure of relative land-use efficiency, can be used to reflect the magnitude of the intercropping advantage (Mohammadkhani et al., 2023). In present study, LER values exceeded 1 under both LP and HP conditions (**Table 6**), indicating that maize has an intercropping advantage, whereas soybean has an intercropping disadvantage in the maize–soybean intercropping system (**Figure 7**), which aligns with previous findings (Yang et al., 2017a). This benefit is attributed to two primary factors: (1) aboveground efficient light interception, where tall maize efficiently intercepts light while shorter soybean utilizes lower-intensity light beneath the canopy (Lv et al., 2014); and (2) belowground complementary resource use and interspecific facilitation, where the fibrous, shallow root system of maize and the taproot, deeper system, and the ability of dinitrogen fixation of soybean reduce direct competition for soil resources, resulting in a more efficient acquisition of nutrients, such as N and P (Dong and Zhang, 2026; Yang et al., 2017b). Indeed, our results support these mechanisms, showing significantly higher N and P accumulation and greater N and P recovery efficiency (ER_N_, ER_P_) in the intercropping system compared with monocultures (**Tables 1**, **5**; **Figures 4**, **5**, **7**). Previous studies on cereal–legume intercropping systems have also reported that intercropping enhances nutrient acquisition, which in turn promotes aboveground growth (Latati et al., 2016; Tariq et al., 2022). Root represents vital factors affecting plant nutrient acquisition. In current study, soybean induced the rapid proliferation of maize roots toward their own root zones (**Tables 3**, **4**, **Figure 6**). The increased root length in surface soil layers enhances maize N and P accumulation, specific N and P uptake, and greater N and P recovery efficiency (ER_N_, ER_P_) in the intercropping system than that of in monocultures (**Tables 1**, **2**, **5**, **6**; **Figures 4**, **5**). Previous studies in maize-soybean intercropping systems have also reported that maize lateral root was expanded into inter-row spaces (Dong and Zhang, 2026), enhancing nutrient acquisition, and maintaining the advantage of intercropping (Latati et al., 2016; Tariq et al., 2022). Notably, the intercropping advantage was more pronounced under LP than HP conditions (**Table 6**; **Figure 7**), suggesting greater relative benefit under phosphorus-deficient conditions. This is consistent with previous studies showing that intercropping provides greater advantages under adverse conditions (Lu et al., 2024; Liu et al., 2026; Song et al., 2026).

Selecting crop species and/or cultivar combinations with traits that maximize positive, and minimize negative, interactions is one of approaches for improving intercropping systems (Brooker et al., 2015). In current study, the benefits of intercropping were modulated by maize root architecture (**Figure 7**). Although intercropping with soybean improved the performance of both the small-rooted (M1) and large-rooted (M2) maize varieties, intercropping of the large-rooted maize variety M2 with soybean showed superior LER, and ER_P_ compared with the small-rooted variety M1 (**Table 6**). This indicates that maize with larger root systems has a greater advantage in nutrient capture in maize–soybean intercropping systems. Results from P accumulation in shoots and roots, and specific P uptake further support this conclusion (**Figure 4**, **Table 5**). The contrasting competitive ability responses between the two maize varieties can be largely attributed to the differences in root morphological traits. The root system is a vital organ for plant nutrient uptake, and its spatial distribution strongly influences nutrient acquisition and, consequently, plant growth (Tian et al., 2026; Zhang et al., 2022). For relatively immobile nutrients, such as P, plants primarily absorb soil P close to the root zone. In the present study, maize varieties with larger root systems indicated greater root spatial overlap with soybean roots, this can be directly observed in the root growth in **Figure 6**. Additionally, the decreased root diameter in the big rooted maize variety when intercropped with soybean was also found, which means more fine roots in the big rooted maize variety (**Tables 3**, **4**). These change of root growth increased the opportunity of maize to capture P from soil and P mobilized by soybean roots, thereby gaining greater benefit from the association. Thess also supported by higher P competition ratio (CR_P_) in the big rooted maize M2–soybean intercropping system (**Table 6**), and indicating stronger P competitiveness of the large-rooted maize variety M2 compared with the small-rooted variety M1, especially under P-deficient conditions (**Figure 7**).

Conversely, for highly mobile N, a large, densely branched root system may intensify intra-root competition, whereas deeper, more sparsely distributed rooting is more advantageous for N acquisition under limiting conditions (Zhan and Lynch, 2015; Postma et al., 2014). Accordingly, the large-rooted variety M2 showed a lower N competition ratio (CR_N_) under LP conditions compared with the small-rooted variety M1 (**Table 6**). Furthermore, intercropping with big rooted maize intensifies interspecific competition between maize and soybean. Soybean is capable of biological N fixation (Dong and Zhang, 2026). When intercropped with large-root maize, the extensive root systems of maize overlap with those of soybean aggravates interspecific competition for the high mobility N fixed by soybean. Therefore, the large-rooted variety did not confer an advantage for N capture (**Figure 4**). Notably, under LP conditions, the increases in LER, ER_N_, and ER_P_ for M2S relative to M1S were more pronounced than those observed under HP conditions. This suggests that under P-deficient conditions, intercropping of large-rooted maize with soybean provides a greater intercropping advantage. Moreover, we also found that soybean also benefited more from association with M2, exhibiting higher biomass and nutrient accumulation than when intercropped with M1 (**Figure 2**-**5**), indicating a mutually beneficial interaction promoted by the large maize root system. However, the current work was carried out in controlled environment, field works in more sites and a broader range of crop varieties should be conducted in the future to verify this result.

An increasing number of studies have reported that appropriately matching growth periods, agronomic traits, and plant types among maize varieties for mixed-cropping is a simple technique that does not increase production costs but can improve both yield and yield stability (Shi et al., 2006; Zhu et al., 2000; Hoekstra et al., 1985a). This is consistent with our results, where mixed-cropping increased N and P accumulation and shoot biomass compared with monoculture (**Table 1**; **Figures 2**-**5**). The specific N and P uptake, LER, ER_N_, and ER_P_ results also showed that mixed-cropping conferred advantages in shoot growth and nutrient acquisition (**Tables 5**, **6**; **Figure 7**). However, some studies in maize have reported that mixed-cropping does not increase yield and may even reduce it (Baker and Briggs, 1984; Hoekstra et al., 1985b). This may be due to the selected varieties exhibiting minimal differences in growth duration and plant architecture, leading to increased competition for above- and belowground resources and, consequently, reduced yield (Shi et al., 2006). We should be notice that the advantages of mixed-cropping were more pronounced under HP than LP conditions (**Figures 2**-**5**; **Table 6**), suggesting that complementary effects in mixed-cropping are enhanced under sufficient P supply. The strong complementary effects in the two maize varieties mixed-cropping systems driven by spatial niche differentiation in root systems (small-rooted and big-rooted maize), allowing efficient nutrient utilization across soil layers and growing season (Du et al., 2020). Previous studies also reported that compared with cereal-legume intercropping, cereal-cereal intercropping systems can provide an optimized option for maximizing plant growth in resource-rich conditions, and higher yield and yield stability finally (Song et al., 2026).

### 4.2 Root distribution influenced by planting methods and P supply

Compared to monocropping, root distribution in soil layers is a key mechanism that increases belowground niche complementarity under intercropping, especially under P-deficient conditions (Bargaz et al., 2017). In the present study, compared to monocropping, three main root-distribution patterns across the intercropped species under P-deficient conditions was found: (1) maize increased RDM, RL, RSA, and RV in the intercropping system. (2) soybean RDM, RL, RSA, and RV were influenced by the intercropped maize varieties. (3) maize increased root growth in deep soil layers as intercrops. The development of roots in the soil was clearly seen in the rhizoboxes (**Figure 6**), where roots of intercropped maize massively spread root underneath the soybean roots and explored a greater soil volume indicating an important intercropping effect in the deeper soil layer. These findings align with previous research indicating that intercropping can modify plant root architecture and spatial distribution (Yuan et al., 2017; Zhang et al., 2016). Similar root distribution has also been reported in the faba bean-maize intercropping systems, that the roots of maize and faba bean intermingle and appear to grow together (Li et al., 2006). Along with this root stimulation, improved N and P uptake, and alleviated of limited nutrient constraints were also reported in cereal rather than legume as intercrops (Yuan et al., 2017; Zhang et al., 2016). Indicating that the complementarity of the spatial root distribution of intercropped species contributes to interspecies facilitation in maize/soybean intercropping compared to the monocropping system.

Analysis of the spatial distribution of RL revealed a noticeable intercropping advantage for the big rooted maize variety as compared to monocropping, that RL in the 0–60 cm soil layers were enhanced. This was especially pronounced in P-deficient conditions (**Table 3**). Previous research also indicated that intercropping can modify plant root architecture and spatial distribution (Yuan et al., 2017; Zhang et al., 2016). As intercropped, big rooted maize variety had higher SDM and RDM, which coincided with improved SNA, SPA, RNA. and RPA (**Table 3**; **Figures 2**, **4**, **5**). Previous studies in maize- faba bean, and maize- chickpea intercropping systems also reported that belowground root growth was significantly correlated to the aboveground acquisition of P and shoot biomass of maize, while no effect was observed with soybean (Xia et al., 2013). This finding indicates that root distribution in soil layer might be important for N and P acquisition in maize supporting productive shoot growth under P-deficiency.

Plant roots respond not only to soil nutrient availability but can also detect neighbouring plants and exhibit adaptive responses (Zhang et al., 2016). Under LP conditions, root morphology in the mixed-cropping system was altered (**Tables 1**, **3**), with responses varying by variety, consistent with genotype-specific recognition and avoidance behaviours reported in other species (Yao et al., 2024). Under HP conditions, phosphorus application mitigated the competitive disadvantage of the smaller-rooted M1, enhancing its root growth and subsequent nutrient uptake in the mixture (**Tables 3**, **4**, **Figure 1**). This suggests that P application can alleviate competitive constraints in mixed cropping, allowing different varieties to more fully express their root system advantages (**Tables 4**, **6**; **Figures 2**, **5**). Studies in cereal mixed-cropping systems have also reported that interspecific competition can be alleviated under improved nutrient conditions, and concluded that the advantage of intercropping is related to crop competitive ability, soil fertility, and fertilizer application (Song et al., 2026). 

## 5 Conclusion

The present study demonstrates that maize-soybean intercropping significantly enhanced maize growth and nutrient acquisition compared with monoculture systems, with the magnitude of these benefits strongly influenced by root architecture and P availability. The large-rooted maize variety M2 conferred a greater advantage, and P uptake in maize-soybean intercropping system, particularly under P-deficient conditions, as indicated by a higher LER and CR_P_. However, this extensive rooting conferred less advantage for N uptake under P deficiency, resulting in a lower CR_N_ compared with the small-rooted variety M1. Mixed-cropping of maize varieties also increased productivity, however, its benefits were more pronounced under high P supply, suggesting that adequate nutrient availability is crucial for realizing the complementary potential of variety mixtures, potentially by alleviating competitive constraints. Overall, root system size and soil P availability jointly determine the outcomes of these cropping systems by modulating belowground competition and complementarity. Given the limitations of the present study, future research should be carried out across more field sites, with a broader range of crop varieties and multi-crop intercropping systems, to further elucidate the effects of root morphology on intercropping.

## Data Availability

The original contributions presented in the study are included in the article/**Supplementary Material**. Further inquiries can be directed to the corresponding author.
