# Supplementary material for: Linking maize root size to phosphorus acquisition and productivity in diversified cropping systems
Source: Front Plant Sci. 2026 Jul 13;17:1876721. doi: 10.3389/fpls.2026.1876721 (PMC13402483; doi:10.3389/fpls.2026.1876721)
Supplement: Supplementary file 1 [file Table1.docx]

Table S1 The rank of maize genotypes is according to the size of root system from big to small. Shengrui 999 (M1) represents small root genotype, and Zhongke 11 (M2) represents big root genotype, but there is a small difference in shoot dry weight between the two genotypes. Soybean seeds were provided by National Center for Soybean Improvement, Nanjing Agricultural University.

| Genotype | Ranking No. | Ranking No. | Ranking No. | References |
| --- | --- | --- | --- | --- |
| Shengrui 999 (M1) | 11 | 121 | 80 | Qiao et al. 2019 |
| Zhongke 11 (M2) | 7 | 8 | 10 | Qiao et al. 2019 |
